# Rescue of degenerating neurons and cells by stem cell released molecules: using a physiological renormalization strategy

**DOI:** 10.14814/phy2.14072

**Published:** 2019-05-02

**Authors:** Greg Maguire, Lee Paler, Linda Green, Rosa Mella, Maria Valcarcel, Patricia Villace

**Affiliations:** ^1^ BioRegenerative Sciences, Inc. San Diego California; ^2^ Auditory Sound Waves, LLC San Diego California; ^3^ Innoprot Derio‐Bikkaia Spain

**Keywords:** Neurodegeneration, neurons, secretome, stem cells, stress granules

## Abstract

Evidence suggests that adult stem cell types and progenitor cells act collectively in a given tissue to maintain and heal organs, such as muscle, through a release of a multitude of molecules packaged into exosomes from the different cell types. Using this principle for the development of bioinspired therapeutics that induces homeostatic renormalization, here we show that the collection of molecules released from four cell types, including mesenchymal stem cells, fibroblast, neural stem cells, and astrocytes, rescues degenerating neurons and cells. Specifically, oxidative stress induced in a human recombinant TDP‐43‐ or FUS‐tGFP U2OS cell line by exposure to sodium arsenite was shown to be significantly reduced by our collection of molecules using in vitro imaging of FUS and TDP‐43 stress granules. Furthermore, we also show that the collective secretome rescues cortical neurons from glutamate toxicity as evidenced by increased neurite outgrowth, reduced LDH release, and reduced caspase 3/7 activity. These data are the first in a series supporting the development of stem cell‐based exosome systems therapeutics that uses a physiological renormalization strategy to treat neurodegenerative diseases.

## Introduction

Physiological renormalization of the immune system instead of enhancement and direct attack is a new strategy in the successful development of recent chemotherapeutics for cancer (Sanmamed and Chen 2018), a strategy for which the 2018 Nobel Prize in Physiology or Medicine was awarded. In other words, strategies of immune normalization therapy instead of enhancement of the immune system or a therapeutic direct attack of the cancer cells, has renormalized T‐cell physiology to perform their normal attack of cancer cells through the B7‐H1/PD‐1 pathway, bringing many new oncology “checkpoint blockade” drugs to the market (Zappasodi et al. 2018). Likewise, renormalization strategies for degenerative diseases, including neurodegeneration, are a new means to return the nervous system to homeostasis, including proteostasis (Maguire 2018a,b), in diseases, such as ALS, that have remained refractory to current therapeutic strategies (Dorst et al. 2018; Maguire 2017). Physiological renormalization is a process that occurs naturally, for example, in sleep‐dependent synaptic downscaling, called synaptic renormalization (Fink et al. 2013). Using a physiological renormalization process to treat diseases may therefore be a therapeutic development strategy that is more efficacious and safer than targeted, genomic approaches that dominate today, and have been overhyped in the USA and elsewhere (Woloshin et al. 2009; Prasad 2016).

As in evolutionary studies, where physiology has returned to the “centre stage” (Noble et al. 2014), therapeutic development must also bring physiology “centre stage” (Moffat et al. 2014) given that genomics centric therapeutic development strategies, having forgotten physiology (Viney 2014), has failed to bring successful therapeutics to the market over the last two decades of genome‐centric research and development programs. In this physiological renormalization strategy, the homeostasis, including proteostasis, of the diseased nervous system is restored through the application of exogenous stem cell released molecules (secretome) in order to provide the molecules, including heat shock proteins (HSP), needed to mimic the functions of the cells and tissues and rebuild the ECM and microenvironment, including the stem cell niche.

Degenerative disorders and neurodegenerative disorders in particular, are one of the most difficult challenges for therapeutic development, medicine, and healthcare because of their poorly understood epidemiology, etiology, pathogenesis, and the heavy burden with which the patients, healthcare system, and society must endure (Dorsey et al. 2013). For example, exposure to multiple toxins that act synergistically to cause neurodegeneration add to the difficulty of understanding and predicting the etiology of a given neurodegenerative disorder (Ku et al. 2016). Our exposome may account for 70–90% of chronic disease (Rappaport and Smith 2010), including the many cases of sporadic neurodegenerative diseases where proteinopathies are greater than previously recognized (Kovacs 2019). Neurodegenerative diseases (NDs) such as glaucoma, sensorineural hearing loss, Alzheimer's disease, and amyotrophic lateral sclerosis among others result from neuronal destruction in the central nervous system. In NDs the volume of the brain is reduced and the number of neurons and other cells decline over time. These diseases reduce the function of patients and destroy tissue and nerves of the brain because neurons in most sections of the brain cannot reproduce themselves, except for selective niches of the adult mammalian brain within areas of the brain such as the hippocampal dentate gyrus (Ming and Song 2005), amygdala (Jhaveri et al. 2017), and the olfactory bulb, although even in the olfactory bulb the regenerative capacity is not limitless (Child et al. 2018). The resident populations of neural stem and precursor cells that proliferate and differentiate into functional neurons, drive neurogenesis in these regions (Kempermann et al. 2018). Neural stem cells also release molecules known to enhance survival of degenerating neurons (Mendes‐Pinheiro et al. 2018).

Many of the neurodegenerative disorders usually occur at an older age, and are characterized by deficits in many cell types and their surrounding tissue, including the extracellular matrix (ECM) and its special condensed form called perineuronal nets (PNN) that surrounds some neurons, especially those with high spike rates (Cabungcal et al. 2013). Stem cell function declines with age, and therefore the ability of stem cells in the brain to make and repair neurons and other tissues, including ECM, of the brain will resultantly decline with age (Conboy et al. 2015).

Maintenance and healing of brain tissue relies on a normal functioning ECM and microenvironment (Maguire 2018a,b), including neural and mesenchymal stem cells that provide maintenance factors, such as chaperones and heat shock proteins (Chiellini et al. 2008). Neurons, once they have become differentiated, do not produce these factors themselves (Oza et al. 2008), and therefore must rely on neighboring stem cells as their source of HSPs. The importance of HSP has recently been demonstrated by Horwich's laboratory where transgenic expression of HSP110 extends the life of mice with a neurodegenerative phenotype, a model that is much like some aspects of ALS (Nagy et al. 2016). Furthermore, other stem cells, such as mesenchymal stem cells, will provide the molecules necessary to build the ECM and microenvironment, and satellite cells in muscle, a type of stem cell will help to regenerate the muscle tissue (Murphy et al. 2011; Maguire 2017). Also, mesenchymal stem cells can protect and rescue the extracellular matrix (ECM), including perineuronal nets (PNN), a condensed form of the ECM, and positively alter the course of neurodegeneration in a mouse model of ALS (Forostyak et al. 2014). Other cells in the brain, including astrocytes, releasing lipoxins, have been shown to be important in rescuing CNS neurons during the degenerative process (Livne‐Bar et al. 2017).

Biology typically constrains intracellular molecules to a particular domain by surrounding the molecules with lipid membranes. However, another mechanism of constraint is present, cytoplasm phase separations (Walter and Brooks 1995; Bowman et al. 2013) such as that induced by the RNA‐binding proteins that sequester transcripts (Hentze et al. 2018). RNA‐binding proteins during oxidative stress events in the cell can bind proteins that are unessential to cell viability, sequestering the unessential proteins and allowing the cell to devote energy and resources to the translation of necessary “housekeeping” proteins. Recent studies have shown that stress granules are assemblies of untranslating messenger ribonucleoproteins (mRNPs) that form from mRNAs stalled in translation initiation. Stress granule assembly minimizes stress‐related damage and promotes cell survival (Mahboubi and Stochaj 2017). Persistent or aberrant stress granule formation, such as FUS stress granules, is associated with neurodegenerative disease and some cancers, and serves as an important biomarker for neurodegenerative diseases (Protter and Parker 2016). FUS is present in exosomes (Kamelgarn et al. 2016), suggesting that the secretion of FUS might contribute to the cell‐to‐cell spreading of FUS pathology in neurodegenerative diseases such as ALS. Because FUS normally regulates microRNA‐based gene silencing, aberrant FUS function can prevent microRNA gene silencing as shown in a model for ALS (Zhang et al. 2018a,b), possibly doing so in a spreading, cell‐to‐cell manner through exosome delivery. Likewise, TDP‐43 stress granule formation is a hallmark of a number of neurodegenerative diseases, such as ALS (Gao et al. 2018).

In this series of studies, we reasoned that neurodegenerative diseases may best be treated with a collective of molecules released from the key cell types in the nervous system that have been shown to rescue neurons. Our reasoning derives from evidence that adult stem cells types and progenitor cells act collectively in a given tissue to maintain and heal organs, such as muscle, through a release of a multitude of molecules packaged into exosomes from the different cell types (Murphy et al. 2011; Fry et al. 2017). Our initial studies therefore use an in vitro model of neurodegeneration, where neurons or U2OS cells were insulted with arsenite or glutamate, and the endpoints measured were stress granule formation, or a number of mechanisms underlying neurotoxicity. Our intervention was to use a proprietary and patented mix of all the molecules in the secretome of four cell types found in brain tissue to determine whether this therapeutic candidate would prevent or lessen the formation of stress granules as an indicator that the intervention prevented oxidative stress in the insulted neurons. The results we present here show a significant reduction in FUS and TDP‐43 stress granule formation, LDH release, and caspase 3/7 activation, along with an increase in neurite outgrowth in the presence of our collective secretome when the neurons were challenged with glutamate. These data suggest that the molecules released from stem cells, and other cell types, provide an important new means to develop systems therapeutics (Maguire 2014) that may provide key advantages over standard small molecule‐targeted approaches (Maguire 2014) and over the use of stem cells themselves (Maguire 2016a,b). Important to our development of a systems therapeutic is allowing the stem cells to release their molecules as opposed to using an extraction process thereby allowing the molecules to fully form and fold correctly, and to package into exosomes. The nanosphere exosome will impart protective, targeting, and penetration qualities to the molecules that would not be present in the molecule's native state without packaging into exosomes (Maguire 2016b).

## Methods

### Cell types and culture methodology

The secretome from four cell types was used to create our therapeutic candidate: 1. Human skin‐derived adipose mesenchymal stem cells (ADSCs) were acquired from ThermoFisher Scientific (Cat. # R7788115), 2. Human skin‐derived fibroblasts (FBs) were acquired from ScienCell (Cat. #2300), 3. Human‐derived astrocytes (Cell Applications, Cat # 882A‐05f), and 4. Human derived immortalized neural stem cells (Millipore, Cat #SCC007). Standard protocols from the manufacturers were utilized, and no antibiotics were used. Cell types and cell culture methodology for the ADSCs and FBs is described in detail in the patents issued to BioRegenerative Sciences (US patent numbers 9545370; 9446075; 20140205563; 20130302273). The secretome from the four cell types, containing both the exosome and the soluble fractions, were combined in equal portions to form the therapeutic candidate; that is, the secretome from each cell type composed one‐fourth of the total collective secretome. Only the secretome from early passages (<10) of the cells was used.

### In vitro testing

**Figure 1:**
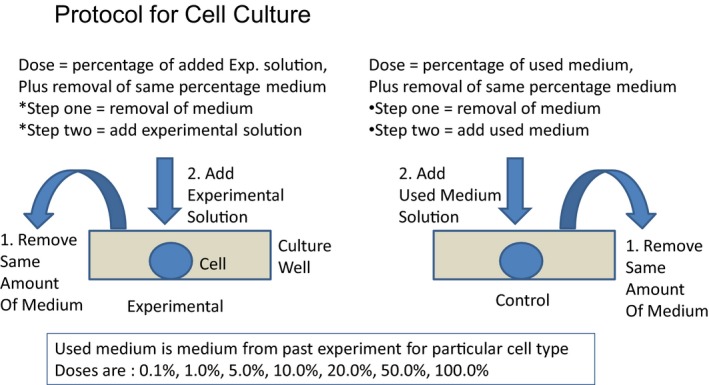
Basic protocol for cell culture in the experimental and control conditions.

A series of three in vitro tests were performed in order to assay the ability of a proprietary mix of secretome from four different types of cells, including stem cells, to rescue neurons from insult due to, (1) Glutamate, (2) Sodium arsenite and the formation FUS stress granules, and (3) Sodium Arsenite and the formation of TDP43 stress granules. All assays were performed in triplicate. In Figure 1 we show the basic protocol for running the cell culture studies.

### FUS study

**Figure 2:**
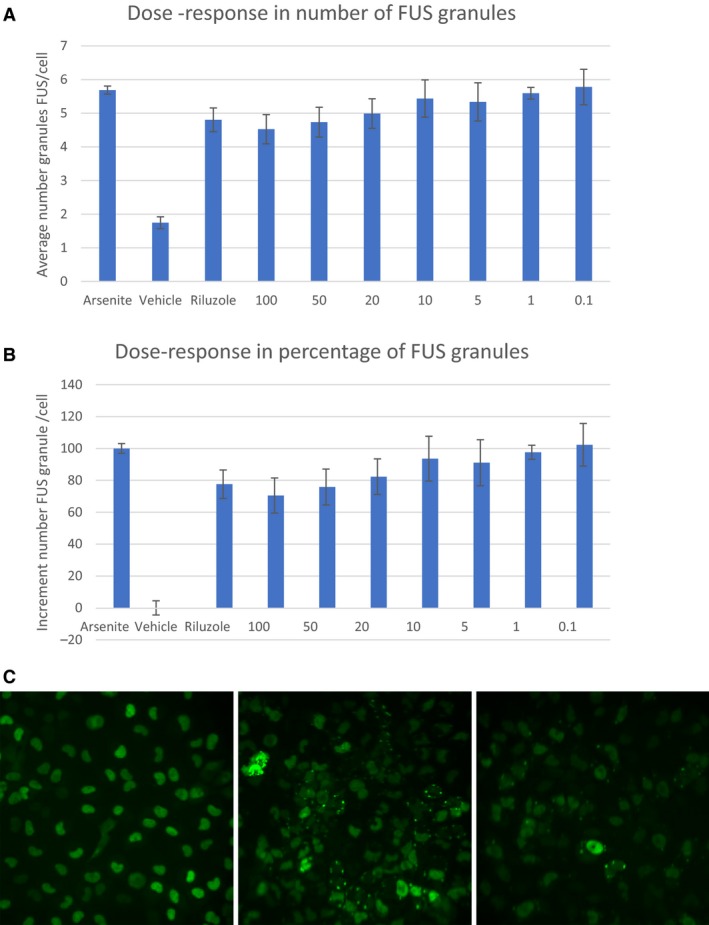
(A) Dose response relationship for experimental secretome at concentrations between 0.1% and 100%. Data points represent the mean ± SD for each condition for a single experiment performed by triplicate. Results are expressed as the average granule number per cell. The images were obtained with an objective of 20 × . 9 pictures of each well were taken. One‐way ANOVA:* P* value 52,637E‐06. (B) Percentage of the FUS granules increment quantification for experimental solution at the concentrations proposed by the Sponsor. Data points represent the mean ± SD at each condition for a single experiment performed by triplicate. The images were obtained with an objective of 20×. 9 pictures of each well were taken. The results were normalized according to sodium arsenite and vehicle, considering Sodium arsenite and vehicle as 100% and 0%, respectively.(C) Representative images. The pictures are representative images corresponding to Vehicle (control), treatment with Arsenite (Ars), treatment with Riluzole at 5 *μ*mol/L concentration, experimental solution provide by BRS at 100% concentration.

**Figure 3:**
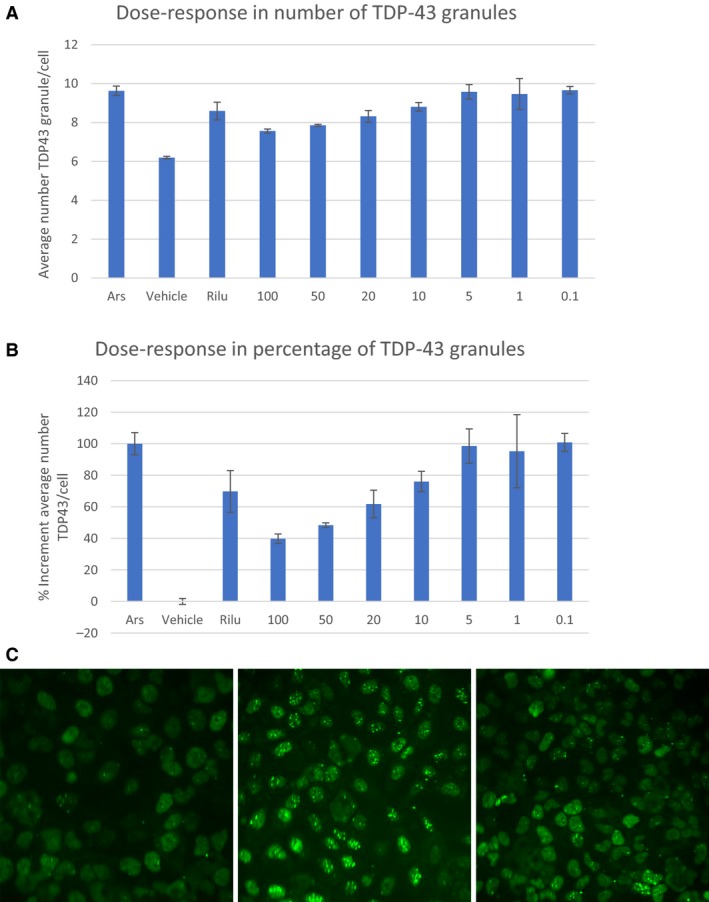
(A) Dose response relationship for experimental solution at the concentrations between 0.1% and 100%. Data points represent the mean ± SD for each condition for a single experiment performed by triplicate. Results are expressed as the average granule number per cell. The images were obtained with an objective of 20×. 9 pictures of each well were taken. One‐way ANOVA:* P* value 1,84,356E‐05. (B) Percentage of the TDP43 granules increment quantification for experimental solution provide by BRS at the concentrations proposed by the Sponsor. Data points represent the mean ± SD at each condition for a single experiment performed by triplicate. The images were obtained with an objective of 20×. 9 pictures of each well were taken. The results were normalized according to sodium arsenite and vehicle, considering Sodium arsenite and vehicle as 100% and 0%, respectively. (C) Representative images. The pictures are representative images corresponding to Vehicle (control), treatment with Arsenite (Ars), treatment with Riluzole at 5 *μ*mol/L concentration, experimental solution at 100% concentration.

Dose–responses for the SRM was performed using a cellular fluorescence redistribution assay after oxidative stress induction in a human recombinant FUS‐tGFP U2OS cell line. The reduction, induced by the compounds, of the FUS stress granules number within the cells after sodium arsenite treatment was measured in triplicate. The FUS‐tGFP stress granules were quantified using the BD Pathway HCS Reader and Attovision Compartmentalization Software. The error bars represent the standard error of the mean for the three replicate wells (Figs. 2 and 3).

### TDP43 study

Dose–response functions for the stem cells released molecules (SRM) were performed using a cellular fluorescence redistribution assay after oxidative stress induction in human recombinant TDP43‐tGFP U2OS cell line. The reduction, induced by the compounds, of the TDP‐43 stress granules number within the cells after sodium arsenite treatment was measured in triplicate. The TDP43‐tGFP stress granules were quantified using the BD Pathway HCS Reader and Attovision Compartmentalization Software. The error bars represent the standard deviation among the three replicate wells (Table 1).

**Table 1:** Summarizing the magnitude of the rescue effects of the experimental Secretome

Compound	0.1% bio	1% bio	5% bio	10% bio	20% bio	50% bio	100% bio
Caspase	0	0	0	2	2	2	2
Max length	0	2	3	2	1	1	2
Root/neuron	3	3	3	3	3	2	3
LDH	2	2	2	2	2	1	0
Total	5	7	8	9	8	6	7
Drug classification	Moderate	Moderate	High	High	High	Moderate	Moderate

Compound	0.1% bio	1% bio	5% bio	10% bio	20% bio	50% bio	100% bio
Drug classification	Moderate	Moderate	High	High	High	Moderate	Moderate

MK801	10 *μ*mol/L
Caspase	3
Max length	3
Root/neuron	3
LDH	2
Total	11
Drug classification	High

### Glutamate neurotoxicity

Glutamate‐induced excitotoxicity in rat cortical neuron (RCN) primary cells was used to assay mitochondrial damage, DNA damage, oxidative stress, neurite outgrowth, membrane integrity measured by LDH release, and apoptosis as measured by Caspase 3 activation. The pattern of neurite outgrowth of the neurons was analyzed by immunocytochemistry against a Tubulin antibody,”TUJ.” MK‐801, a glutamate receptor (NMDA) antagonist was used as a positive control. For determining plasma membrane integrity, the supernatants were collected 24 h after treatments and an LDH assay was performed following the instructions of the kit manufacturer (Roche).

Our specific endpoints for glutamate toxicity were the following:

1. Apoptosis: Caspase‐3 activation for Apoptosis
2. Neurite Outgrowth: Tuj for Neurite Outgrowth
3. Neuronal Viability: Cell Counting as viability (Hoescht and WST‐8 assay)
4. Membrane Damage: LDH Release (Smith et al. 2011)

### Protocol

Cortical neurons from embryonic 18 days rats were plated in poly‐l‐lysine coated 96‐well plates with a number of 30,000 cells per well. Cells were maintained in neurobasal medium supplemented with B‐27 component for 8 days at 37°C in a humidified 5% CO_2_ atmosphere. At day 8, cells were pretreated with seven increasing concentrations of the conditioned media for 1 h in neurobasal medium supplemented with B‐27 component, washed, and returned to drug‐free medium for up to 48 h before being subjected to a glutamate excitotoxicity condition where cells were incubated with 100 *μ*mol/L glutamate during 15 min in medium without B‐27 component. After glutamate exposure, medium was replaced with neurobasal medium with B27 factor, and then cells were incubated for an additional 24 h. Some cells were treated as described above with MK801 10 *μ*mol/L as positive controls of neuroprotection.

### Statistics

A one‐way ANOVA was applied in each experiment with eight conditions, each condition reflecting a different concentration of the experimental secretome, from control to 100% concentration.

## Results

For the three different studies (FUS, TDP43, and glutamate neurotoxicity), each showed a significant reduction in neuronal insult when the experimental arm was compared to controls.

### FUS

Seven concentrations of SRM were examined for their inhibitory effects on FUS granules formation after cytotoxic stress induction by arsenite. The number of stress granules containing FUS per cell was analyzed for each compound.

SRM was positive in the inhibition of FUS granule formation after arsenite treatment. SRM‐induced stress granule inhibition in a dose‐dependent manner with a maximum of 30.5% at 100% of the concentration, and the effect was no longer observable at a concentration of 20% or less.

### TDP43

Seven concentrations of SRM were examined for their inhibitory effects on TDP‐43 granules formation after cytotoxic stress induction by arsenite. The number of stress granules containing TDP43 per cell was analyzed for each compound.

SRM was positive in the inhibition of TDP43 granule formation after arsenite treatment. SRM‐induced stress granule inhibition in a dose‐dependent manner with a maximum of 60.2% at 100% of concentration, and the effect was no longer observable at a concentration of 10% or less.

### Glutamate neurotoxicity

**Figure 4:**
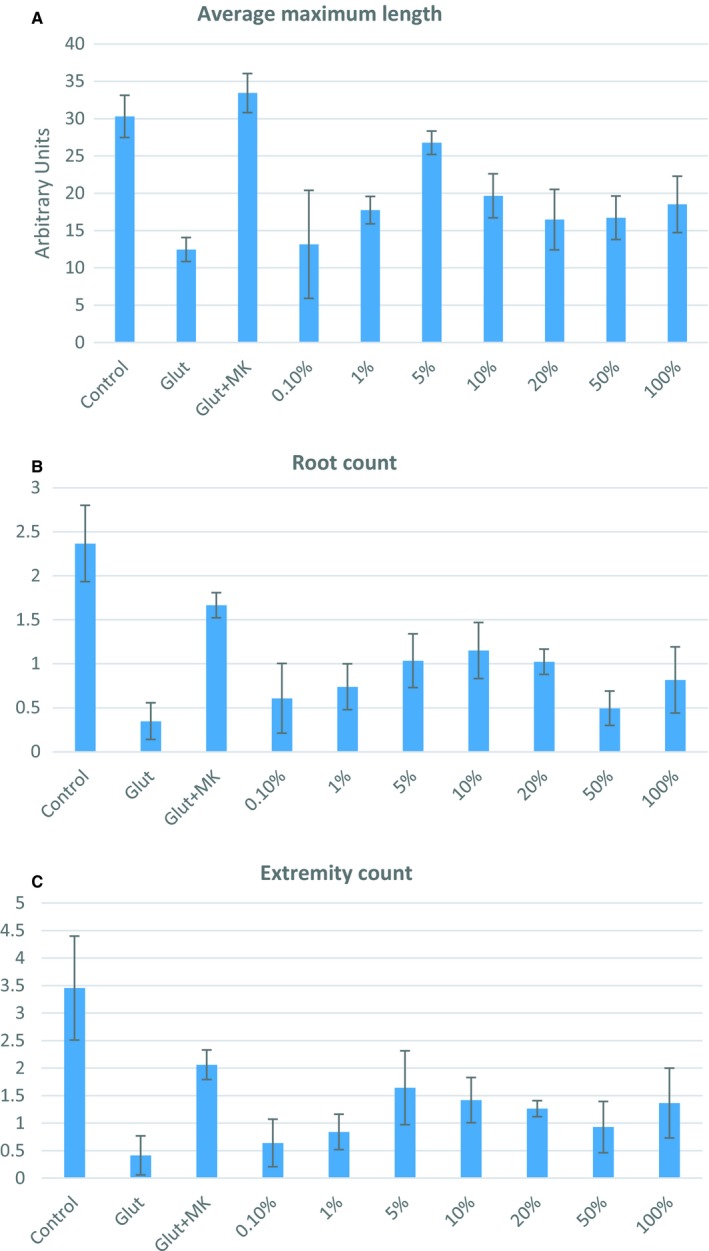
(A) Maximum length of neurite outgrowth as a function of glutamate toxicity and rescue from toxicity with experimental secretome at concentrations between 0.1% and 100%. (B) Mean neurite root count as a function of glutamate toxicity and rescue from toxicity with experimental secretome at concentrations between 0.1% and 100%. (C) Mean neurite branch (extremity) count as a function of glutamate toxicity and rescue from toxicity with experimental secretome at concentrations between 0.1% and 100%.

Neurite outgrowth was measured as an indicator for rescue from glutamate neurotoxicity. As shown below, we measured three different parameters of neurite outgrowth to demonstrate that the experimental secretome significantly rescued neurons from glutamate neurotoxicity. In Figure 4A is shown the average maximum length for neurites for the different cell culture conditions. Compared to controls where glutamate was shown to significantly reduce neurite length, 5.0% of the experimental secretome significantly enhanced the length of neurites.

Another parameter for measuring neurite outgrowth is the number of neurite roots extending from the neuron's soma. In Figure 4B is shown the mean number of roots counted under the different experimental conditions. Compared to the control condition where the neurons were exposed to glutamate, the experimental secretome at a concentration of 10% significantly increased the number of neurite roots.

The number of branches on the roots of the neurites was another parameter we measured as an assay for neurotoxicity. In Figure 4C we show that glutamate significantly reduces the number of branches, and that the experimental secretome significantly increases the number of branches compared to the control glutamate condition. The rescue of number of branches was maximal at a concentration of 5.0% experimental secretome. Higher concentrations of the experimental secretome were not as effective in rescuing neurite outgrowth, likely because the diluted culture medium did not supply all of the requisite factors needed for optimal neurite growth.

### Glutamate neurotoxicity – caspase activation

Caspase activation levels were measured in primary rat cortical pyramidal neurons as an indicator for rescue from glutamate neurotoxicity. Caspases are a family of endoproteases important for maintaining homeostasis through regulating cell death and inflammation. In primary cortical cells, glutamate‐induced cell death occurs through upregulation of caspase‐3 and its activation of a caspase‐dependent pathway involving mitochondrial signaling (Zhang and Bhavnani 2005). We therefore measured caspase levels in cortical neurons challenged by glutamate to determine whether our collective conditioned media could inhibit caspase‐3 and ‐7 levels. At concentrations of 10% and higher, our collective secretome significantly reduced the levels of caspase 3/7 compared to those levels induced by overexposure to glutamate, and even reduced the caspase 3/7 levels below that of the control condition where no glutamate was applied.

**Figure 5:**
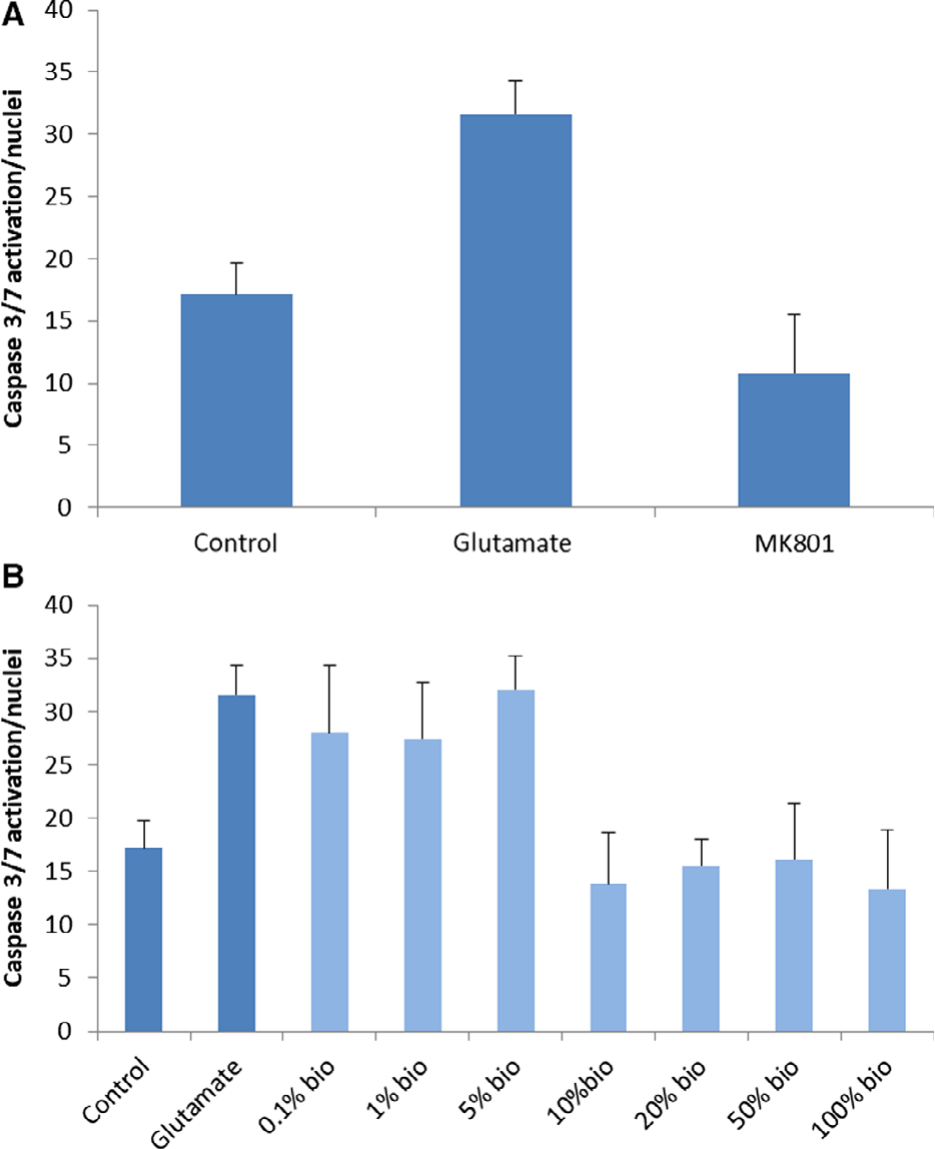
(A) Validation of caspace 3/7 activity in control conditions, glutamate exposure, and the antagonism of glutamate by MK801. This study validated that glutamate induces an increase in caspase 3/7 activity in cortical neurons and that MK801, a glutamatergic NMDA receptor antagonist, blocked the effects of glutamate on the induction of caspase 3/7 activity. (B) Caspase 3/7 activity in primary cortical neurons exposed to glutamate in combination with the S2RM collective secretome. The control shows the baseline level of caspase activity without exposure to glutamate or the S2Rm. The glutamate bar is for exposure to glutamate without S2RM. The bio bars indicate exposure to glutamate plus the addition of the various percentages of the S4RM‐N secretome at concentrations from 0.1% to 100%. Each condition was run in triplicate. These data show that concentrations of the S2RM collective secretome at 10% and higher significantly reduced the induction of caspase 3/7 activity in cortical neurons.

Glutamate treatment resulted in a twofold increase of caspase 3/7 activation in cortical neurons when measured 24 h after exposure at 100 *μ*mol/L concentration, and positive control MK801 prevented glutamate‐induced apoptosis by 65% (Fig. 5). Glutamate‐induced caspase 3/7 activation was also prevented by application of the highest concentrations of S4RM‐N (10, 20, 50, and 100%). Therefore, S4RM‐N secretome supplementation significantly reduced caspase 3/7 activation and improved the survival of cortical neurons.

### LDH‐based cytotoxicity

**Figure 6:**
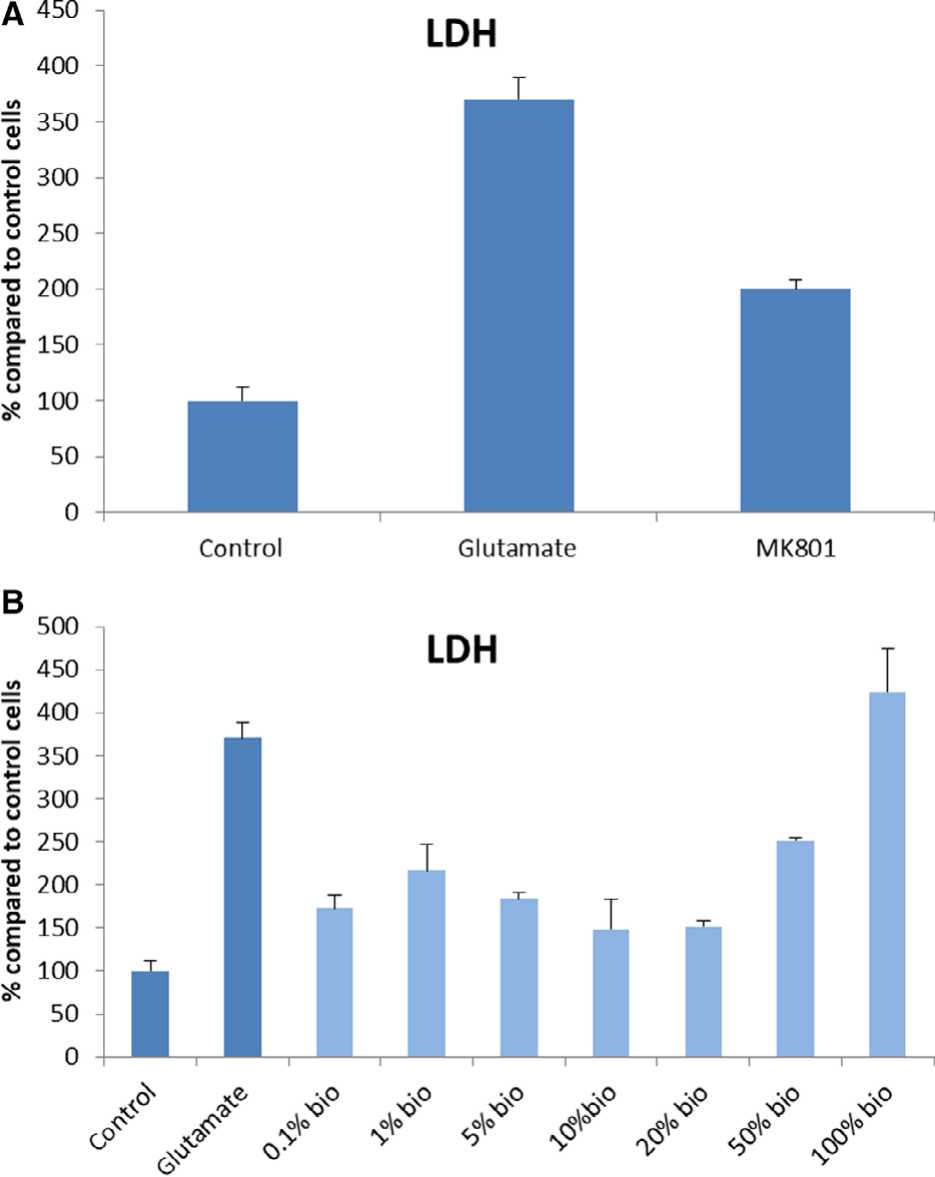
(A) LDH secretion determination using positive and negative control. Neurons were pre‐treated with MK801 10 *μM* in complete medium for 1h and then returned to drug‐free complete medium for 48h. Cultures were then exposed to glutamate 100 *μM* for 15 min and 24 h later LDH assay was performed. (B) LDH secretion in cells treated under glutamate excitotoxicity condition. Neurons were pre?treated with increasing concentrations of the compound in complete medium for 1h and then returned to drug‐free complete medium for 48h. Cultures were then exposed to glutamate 100 *μM* for 15 min and 24 h later LDH assay was performed. Data points represent the mean ± SD for each condition. The results of the compounds were normalized according to the control cells. “Bio” indicates the concentration of the experimental secretome added to the culture dish.

To assess the effects of glutamate on plasma membrane integrity, LDH quantification in supernatants of treated cells was employed. Glutamate induced a 270% increase of LDH in cultured cortical neurons, while the positive control using MK801 normalized the plasma membrane integrity by 45% (Fig. 6). Glutamate induced cell death was also prevented by application of all concentrations tested with the exception of 100% concentration. Therefore, the test therapeutic (S4RM‐N secretome) significantly reduced LDH release and improved survival of the cortical neurons. Different grades of neuroprotection were observed with each compound at the different concentrations tested (Fig. 6).

We summarize the protective effects of S4RM‐N secretome in the glutamatergic neurotoxicity studies as follows. For the therapeutic secretome (S4RM‐N) studied under the various parameters, a variation of at least 20% in fluorescence intensity or in the corresponding morphological parameter in relation to untreated cultures was established. In order to compare the degree of neuroprotection, the level of change for each parameter at 24 h was studied at each concentration. Four different scores of neuroprotection were established according to the level of variation when compared with control cells: 0 (no neuroprotection or variation lower than 20%), 1 (variation 20–40%), 2 (variation 40–60%), and 3 (variation > 60%). The sum of each individual score resulted in the total level of neuroprotection for each compound and was defined as its degree of neuroprotection. From this calculation, a neuroprotective scale was established: high (>8), moderate (5–7), low (1–4), and no neuroprotection (0).

In the present study glutamate toxicity was linked to an increase in caspase 3/7 activation, LDH secretion, and decreased neurite outgrowth. The preventive effects of S4RM‐N against glutamate toxicity are associated with restoration of caspase 3/7 activity, stabilization of neurite outgrowth, and decrease in LDH secretion. All concentrations tested of S4RM‐N scored an optimum degree of neuroprotection level and was shown to be an efficient strategy for the treatment of glutamate toxicity. Neuroprotection from glutamate toxicity was most efficacious at the concentrations of 5, 10, and 20% compared to the other concentrations.

## Discussion

Our data show that molecules released from a collective of four cell types known to be important to neuronal function and regeneration can rescue isolated neurons from glutamate insult, and rescue U2OS cells from arsenite insult as measured in vitro. Specifically, the secretome from neural stem cells, mesenchymal stem cells, astrocytes, and fibroblasts was able to mitigate FUS‐ and TDP‐43 stress granule formation in U2OS cells, and a number of key mechanisms underlying glutamate neurotoxicity in CNS neurons, including : 1. Mitochondrial function, 2. Neurite outgrowth, 3. Membrane integrity, 4. Neuronal viability, and 5. Apoptosis.

Our methodology for therapeutic development depends on targeting pathways at multiple levels of the system, including protein and genomic levels. Considering the protein‐level pathways, many natural molecular, cellular, and tissue functions are initiated and maintained by protein‐level circuits. As an example, caspase mediated programmed cell death, apoptosis, is orchestrated by a circuit of proteases that activate one another by cleavage (Budihardjo et al. 1999). Modifiable protein circuits offer a number of advantages over genetic circuits, including faster operation, direct coupling to endogenous pathways, single‐transcript delivery, and function without genomic integration (Gao et al. 2018). Indeed, protein‐level therapeutics may be very important to neurodegenerative diseases because the disease state may often occur at the protein level, not the genomic level (Maguire 2017). In this regard, Horwich's laboratory at Yale showed that in an animal model of ALS, although genomic correlates have been found to the disease, transcripts were found to be normal, suggesting that the disease state occurs at the level of protein translation or posttranslation despite the association of some genetic defects (Bandyopadhyay et al. 2013).

Inhibiting stress granule number, as is shown in this study, has been shown to diminish nucleocytoplasmic transport defects as well as neurodegeneration in C9ORF72‐mediated ALS/FTD model (Zhang et al. 2018a,b). The formation of these stress granules is context dependent, and the content of the stress granules reflects that context (Markmiller et al. 2018). This means the assemblies of proteins that condense in tight clusters to interact with RNA when cells respond to cellular stresses are different depending on the type of stress experienced by the cell. People with neurodegenerative disease may have an aberrant formation of stress granules (Markmiller et al. 2018) depending on factors from the patient's exposome, phenotype, genotype, or structural cross‐seeding mechanisms in the self assembly of proteins (Nizynski et al. 2018). When stressors are removed, many stress granules disassemble, but a significant proportion relies on autophagy for their elimination (Buchan et al. 2013). The mechanisms by which our secretome reduced the number of stress granules may relate to the prevention of stress granule formation or to their enhanced removal by disassembly or autophagy. Because RNA has recently been shown to have chaperone activity and help to fold proteins (Docter et al. 2016), aberrant stress granule formation that may bind RNA chaperones could be a factor in causing protein misfolding and their self‐templating and prion‐like spreading in neurodegenerative diseases (Goedert et al. 2010). This prion‐like, protein‐only somatic inheritance may contribute to many diseases, including cancer phenotypes given that p53, a protein with the task of suppressing tumor formation in the body, may show a typical prion‐like behavior when mutated (Bom et al. 2012).

Mitochondrial dysfunction is common to most neurodegenerative diseases, including ALS, Alzheimer's, Parkinson's (Smith et al. 2017), glaucoma (Lee et al. 2012), and possibly sensorineural hearing loss (Kaheel et al. 2018). Our study measured TDP‐43, known to bind and regulate the processing of transcripts encoding mitochondrial proteins (Izumikawa et al. 2017), within stress granules and found that our experimental secretome reduced the amount TDP‐43 in the granules compared to control cells.

Glutamate is the most prominent excitatory transmitter in the nervous system, and under normal conditions the concentration in interstitial tissue is well regulated by glutamate transporters that serve to transport glutamate from the extracellular to the intracellular compartments. However, under conditions that mimic neurotoxicity, the transporter can reverse (Szatkowski et al. 1990; Grewer et al. 2008) leading to increased glutamate accumulation in the extracellular compartment of the CNS (Maguire et al. 1998). An increased concentration of extracellular glutamate in the nervous system and the ensuing toxicity are thought to partially underlie a number of neurodegenerative diseases (Lewerenz and Pamela Maher 2015). In our studies we have shown a direct effect of the experimental secretome on neurons to block glutamate toxicity. The direct effects of the secretome can be through many mechanisms, including the induction of neuroglobin (Baez‐Jurado et al. 2018) to reduce ROS production, subsequently upregulate membranous Atp1b1, suppressing the glutathionylation of Atp1b1, and eventually preserving the activity of proteins such as NKA (Wen et al. 2018), involved in pumping ions across the membrane and assembly of protein complexes (Cui and Xie 2017).

Another possible mechanism for the rescue of cells by our collective secretome is through protection of the stressed cell's proteins is by heat shock proteins (HSPs) contained in the secretome of stem cells (Chiellini et al. 2008; Teixeira et al. 2015; Nie et al. 2018), and known to be contained in exosomes. Neurons are particular vulnerable to stress because they do not make their own HSPs (Oza et al. 2008). As a result, stressed neurons not in contact with surrounding stem cells and the HSPs that are supplied by the surrounding stem cells, are at risk. However, neurons have been shown to be rescued using exosomes (Jarmalavičiūt≐ et al. 2015) released from support cells that contain chaperone proteins (Sreekumar et al. 2010). Our study is similar, having used a collective secretome, known to contain exosomes (Maguire et al. 2013) and thought to contain HSPs (Maguire 2017).

In studies where indirect effects of the secretome can be measured, unlike our isolated primary neurons in the present study, we would expect additional efficacy because the ECM and the microenvironment (ECM‐M) surrounding the neurons is built and repaired from the molecules in the secretome, including matrix proteins, and the ECM‐M may be critical to regulating glutamate toxicity and hyperexcitability (Chen et al. 2008; Frischknecht et al. 2009; Vedunova et al. 2013). The importance of a well maintained ECM‐M is fundamental, even at the level of regulating stem cell function, where a disrupted stem cell niche may profoundly alter the stem cell's gene expression profile (Van Velthoven et al. 2017). Indeed, adult stem cells sense their environment and develop epigenetic memories of the event that persist for long periods (Naik et al. 2018). The rebuilt ECM‐M may also facilitate the formation and function of tunneling nanotubes (Osteikoetxea‐Molnár et al. 2016), allowing the stem cells to supply molecules and even organelles, such as mitochondria, to their surrounding neurons in need of rescue (Wang and Gerdes 2015). Furthermore, because the immune system, including T‐cell regulation plays a large role in neurodegeneration, including glaucoma (Chen et al. 2018a,b) and multiple sclerosis (Haase et al. 2018), the immune modulatory capacities of MSCs are suggested by the inhibition of T‐ and B‐cell proliferation (Duffy et al. 2011; Franquesa et al. 2012), inhibition of the production of H_2_O_2_ from neutrophils (Raffaghello et al. 2008), and T and NK cytotoxicity (Spaggiari et al. 2008), as well as suppression of the differentiation and maturation of monocytes into dendritic cells (Ivanova‐Todorova, 2009).

The immune system, acting though T cells, has now been shown to interact with resident HSPs in the resident neural tissue undergoing neurodegeneration (Chen et al. 2018a,b). T cells, conditioned by bacteria in the gut, can invade neural tissue once thought to be immunoprivileged. In the case of a glaucoma model, elevated intraocular pressure upregulates membrane bound and extracellular HSPs in the surrounding neural tissue, and allows T cells to enter the neural tissue compartment because of a compromised blood‐retinal‐barrier (Flammer et al. 2002). Because HSPs are so well conserved from bacteria to mammals, T cells conditioned by HSPs in gut bacteria will attack the mammalian HSP because of molecular mimicry between, for example, bacterial HSP65 and human HSP65 (Rajaiah and Moudgil 2017). Once the HSPs in the neural tissue are compromised by the invading T cells, neurons in that tissue may degenerate in a spreading fashion (Lackie et al. 2017). Thus infusion of normal, exogenous HSPs instead of indogenous, malformed HSPs, into degenerating neural tissue may be an important therapeutic strategy in general, and for ALS and glaucoma specifically (Maguire 2017).

Without consideration of glutamatergic mechanisms, we would expect the efficacy of our experimental secretome to be greater within intact tissue because our mixture includes molecules that should work well by indirect mechanisms, that is, not directly on the compromised cells themselves, rather working on other cell types to activate a number of pathways leading to a number of therapeutic events such as the rebuilding of stem cell niches and the microenvironment/ECM of the affected tissues (Maguire 2017, 2018a,b). In a state of dynamic reciprocity (Bissell and Aggeler 1987), the ECM acting through a number of mechanisms, including mechanical forces at chromatin (Maniotis et al. 1997), may activate stem cells to genetically reprogram themselves to rebuild tissue (Ransom et al. 2018). Essentially a reversion to a more primordial, developmental state in adult stem cells underlies new tissue growth. Other approaches to ameliorating neurodegeneration involve implantation of stem cells, including neural stem cells. Recent studies show that transplanted neural stem cells derived from iPSCs are able to engraft into injured spinal cords and form synapses (Kumamaru et al. 2018). Engraftment of stem cells, whether they are autologous or from a different patient, may have the unintended consequence of inducing aging of the tissue as measured by a p16 biomarker (Wood et al. 2016). While neural stem cell transplantation is an important approach for neuroregeneration, there are many other cells and tissues involved in the repair process in addition to the neural stem cells. As such, the regenerative therapeutic candidate needs to be considered as acting within the system, and address repair of the other cells types. In neurodegenerative indications such as trauma to the spinal cord, cellular transplants will likely be required in order to reconnect the two distal ends of the truncated tissue, that is, spinal cord. However, in other neurodegenerative indications, such as neurodegenerative diseases, for example ALS and Alzheimer's, physical truncation of tissue, including nerves, will be less severe or nonexistent. In these cases, especially with early detection strategies, enabling repair of the neurons and other tissues in the nervous system may be best facilitated with the molecules that stem cells release (Maguire 2016a,b, 2017). In this manner, repair of the system, not just one cell type, can be best addressed, facilitating repair of all the neural tissue.

Stem cells are known to release many of the members of the insulin‐like growth factor‐binding protein (IGFBP) family (Park et al. 2010). Yamahara et al. (2018) have recently shown that exogenously applied IGF‐1 alone can modestly mitigate the effects of traumatic mechanical insult during cochlear implant in a guinea pig model. A slight decrease in thresholds for auditory brainstem responses was measured at low‐frequency stimulation when IGF‐1 was administered compared to controls in which saline was used. Reductionist approaches using one growth factor or a few growth factors, such as NGF (Aloe et al. 2015) in an attempt to prevent or mitigate neural damage has been widely used for years with poor results, including poor results in the treatment of ALS (Petrou et al. 2016). Rather, a wide variety of factors, secreted from multiple cell types, including lipids secereted by glia (Livne‐Bar et al. 2017), microRNA (El Fatimy et al. 2018), and proteins (Gumy et al. 2010), likely play a role in forming a collective secretome that can more completely rescue neurons and neural tissue during various conditions such as ALS (Maguire 2017). The approach we use therefore is called homeostatic renormalization, where not only proteostasis is achieved with administration of the therapeutic but also renormalization of homeostasis for lipids (Livne‐Bar et al. 2017) and microRNA (El Fatimy et al. 2018; Rizzuti et al. 2018) for example. Future studies will need to tease apart the factors in our collective secretome responsible for rescue, including the factors contained within the exosomes and within the secretome solution, and those factors that may not be involved in rescue (Maguire 2018a,b).

### Organizing principles for stem cell function

Reductionists strategies still dominate the field of therapeutics, including stem cell therapeutics, where, for example, “Determining the most appropriate cell type and tissue source is crucial for successful clinical translation of cell‐based therapies” is still the thinking (Chaubey et al. 2018). Instead, we should first recognize that there are many distinct adult stem cell phenotypes, each phenotype optimized for the particular tissue in which it operates, with more than one stem cell phenotype operating within a given tissue. Bone marrow stem cells are distinct from neural stem cells for example, and each cell type will release a pool of 100s of molecules that act collectively in a synergistic manner to promote homeostasis, such as proteostasis and cellulostasis (cell survival), for example (Nie et al. 2018).

Different cell types, including adult stem cells, are known to secrete unique pools of proteins and other molecular constituents (Statna and van Eyk 2012; Berardis et al. 2014). We suggest that one or the organizing principles in stem cell function is that the more differentiated the stem cell, the more specialized will be the SRM from the stem cell (Maguire 2018a,b). This means that embryonic stem cells will release a more general set of molecules than will adult stem cells. This also means that stem cells in a given tissue, even within subregions of a given tissue, will release tissue‐specific molecules. A bone marrow stem cell, for example, will not release the same set of molecules as does a neural stem cell. Each stem cell, progenitor cell, or other cell type within a given region of tissue will release a given set of molecules to maintain or renormalize homeostasis in that given tissue.

A hierarchical organization of stem cells and progenitors cells has been demonstrated in some tissues. Stem cells within a tissue, intrinsic stem cells, act on intrinsic progenitor cells. Such a hierarchy has been demonstrated in the skin where intrinsic stem cells, called keratinocytes (Fuchs 2018), are able to control fibroblasts (Ghaffari et al. 2009) and other cells through the release of exosomes (Cicero et al. 2015). A similar hierarchical organization has been demonstrated in muscle that facilitates muscle regeneration (Fry et al. 2017). Also, bone marrow stem cells (BMSCs) that circulate through the body within the blood may be considered intrinsic under certain conditions, such as wounding. Normally these bone marrow stem cells are not found in tissues such as the skin or brain, but upon demand, during injury will be recruited to the damaged tissue, including the brain during neurodegenerative events (Kierdorf et al. 2013). The purpose for recruiting the BMSCs maybe that the intrinsic stem cells do not secrete GDF‐11 (Lee et al. 2010), whereas the BMSCs do secrete GDF‐11 (Lai et al. 2010). This is important because GDF11 will inhibit proliferation and drive differentiation of stem cells (Williams et al. 2013). Essentially this is a maturation effect that is required to form new somatic cells to replace the injured cells. Careful recruitment of BMSCs only for injury is important so that constant differentiation and lack of proliferation does not exhaust the intrinsic stem cell supply and therefore prematurely age the tissue.

In conclusion, our studies show that the collective conditioned media from four cell types intrinsic to neural tissue directly rescue neurons and cells from stress and glutamate‐induced neurotoxicity. We also expect that paraneurons, such as auditory hair cells (Géléoc and Holt 2014) and retinal photoreceptors that, under some conditions in humans, may be able to regenerate using our strategy (Horton et al. 2015). We also expect, and have experiments underway to determine the effects of this collective conditioned media on neurons and other cells through indirect mechanisms, such as the rebuilding of the ECM/microenvironment, basement membranes, and the stem cell niches within the neural tissue. Although our initial study here used the collective secretome from four cell types demonstrated to be relevant to the nervous system and to neural repair, future studies will need to explore two key areas: (1) tease apart whether all components are necessary for the observed effects, or whether a subset of the four components used are sufficient to observe the therapeutic effect, and (2) we have also begun mass spectrometry studies to understand the molecular components our collective secretome, and metabolite and lipid analysis will also be necessary to fully characterize the content, including microRNA species known to be important to homeostasis of the nervous system (Pegtel et al. 2014). Understanding these two areas may lead to an approach where a reductionist set (system) of molecules, the minimum molecule set (MMS), that is not overly reductionist so as to be ineffective, but instead uses the least number of necessary molecules that are sufficient to realize a safe and efficacious MMS therapeutic (Maguire 2014), can be used to treat neurodegenerative diseases.

## Conflict of Interest

None declared.
